# Case Report: Hepatopulmonary fusion: to separate or not to separate? From a clinical case to A literature review

**DOI:** 10.3389/fped.2025.1497203

**Published:** 2025-03-25

**Authors:** Marta Tedesco, Simonetta Costa, Pierpaolo Agresti, Francesca Priolo, Alessandro Perri, Annamaria Sbordone, Stefano Nobile, Filomena Valentina Paradiso, Maria Vittoria Stern, Riccardo Rizzo, Maria Cristina Giustiniani, Lorenzo Nanni, Giovanni Vento

**Affiliations:** ^1^ Neonatal Intensive Care Unit, Department of Woman and Child Health and Public Health, Fondazione Policlinico Universitario Agostino Gemelli IRCCS, Rome, Italy; ^2^ Catholic University of Sacred Heart, Rome, Italy; ^3^ Pediatric Surgery, Department of Woman and Child Health and Public Health, Fondazione Policlinico Universitario Agostino Gemelli IRCCS, Rome, Italy; ^4^ Department of Pathology, Fondazione Policlinico A. Gemelli IRCCS, Rome, Italy

**Keywords:** hepatopulmonary fusion, congenital diaphragmatic hernia, right-sided congenital diaphragmatic hernia, management, neonatal intensive care unit

## Abstract

**Objective:**

Hepatopulmonary fusion (HPF) is a rare congenital malformation, frequently associated to right-sided congenital diaphragmatic hernia (CDHR). The presence of HPF often leads to a fatal outcome. The most effective approach to managing this condition remains uncertain due to the limited number of documented cases in the literature.

**Study design:**

This case presents a 11-day old full-term female neonate with HPF associated to CDHR. The definitive diagnosis of HPF was made during surgery for CDHR. Our team opted for a simple repair of the diaphragmatic defect and no attempts were made to separate the liver from the right lung.

**Results:**

Our approach was successful, as our patient not only survived the procedure but also showed favorable cardiorespiratory adaptation, consistent growth, and regular neurodevelopment, according to follow-up data, available at six months of life.

**Conclusion:**

The adopted surgical management strongly suggests that when the diagnosis is made intraoperatively and detailed knowledge of the vascularization is lacking, partial separation of the viscera, preserving the medial hepatopulmonary fusion and suturing the diaphragm, is the successful approach.

## Introduction

Congenital diaphragmatic hernia (CDH) is a rare congenital malformation, with an estimated incidence of 2.4–4.2 per 10,000 births in the world, with right-sided CDH (CDHR) being the rarest form, accounting for approximately 15% of cases of diaphragmatic hernia (1). In comparison to left-sided lesions, the prognosis for CDHR is generally worse (2).

Hepatopulmonary fusion (HPF) is a rare congenital malformation associated with CDHR. Its prevalence is approximately 3 in 1,000 newborns affected by CDHR, and it affects both sexes equally. The anomaly can encompass a spectrum of fusion levels, spanning from fibrovascular connections to the complete merging of the pulmonary and hepatic tissues (3, 4). The presence of HPF often leads to a fatal outcome when combined with CDHR (5). The overall mortality associated with HPF or its complications is around 49% (6).

Due to the rarity of this condition, there is a lack of well-established guidelines for the optimal management strategy for HPF. In this study, we present a case of successful management of CDHR accompanied by HPF and provide an extensive review of the existing literature.

## Clinical report

A female infant was born at 37 weeks and 3 days of gestational age (GA) from an uneventful pregnancy, with a birth weight of 2,370 g (small for GA, 1.39 z-score, according to Intergrowth-21). No clinical problems occurred at and after birth, and the baby was discharged after 3 days of Rooming-in. At 5 days of life, the infant experienced mild respiratory distress, which required an outpatient visit to the attending pediatrician. Upon examination, the pediatrician observed tachypnea and performed blood tests for acid-base balance and C-reactive protein, both of which yielded negative results. At 11 days of life, the baby was admitted to the emergency room due to a sudden episode of apnea during feeding. Soon after the arrival, the newborn appeared pale and hypotonic, with no respiratory activity and an oxygen saturation of 73%. Blood gas analysis revealed severe respiratory acidosis (pH 6.9, pCO_2_ 114 mmHg, lactate 10 mmol/L, base excess −10 mmol/L). Therefore, the baby was intubated, and ventilation was continued with 100% FiO_2_. Subsequently, she was transferred to our Neonatal Intensive Care Unit (NICU) with a suspected diagnosis of aspiration pneumonia.

**Figure 1:**
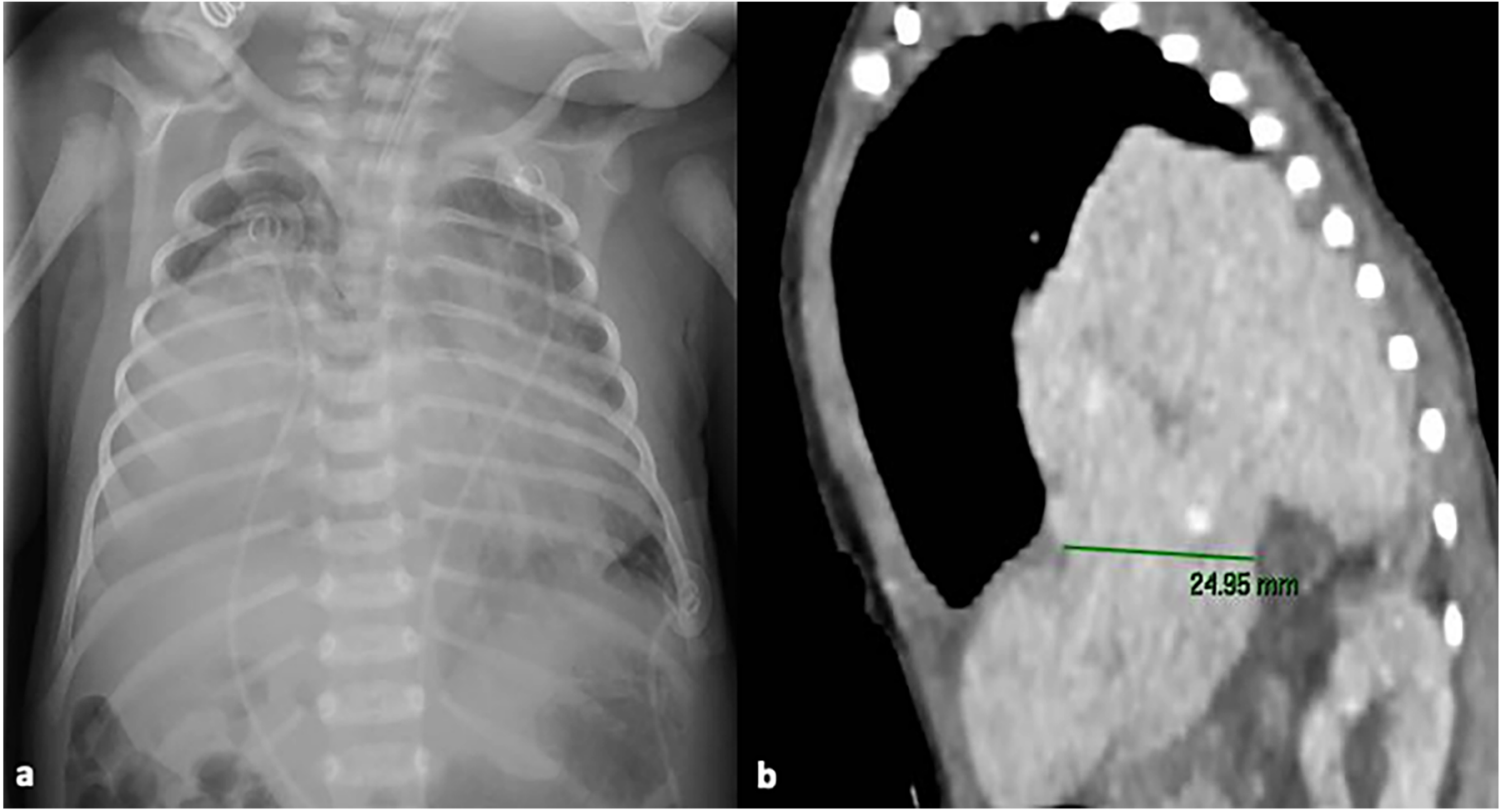
Chest x-ray at NICU admission showing areas of consolidation in both lung fields **(a)**; chest computed tomography scan, showing the presence of a posterior CDHR, through a diaphragmatic defect measuring approximately 25 mm: the liver was herniated into the intrathoracic space, reaching up to the middle-upper third of the right lung field **(b)** written informed consent was obtained from the patient.

Upon admission to the ward, a chest x-ray was performed, which revealed areas of consolidation in both lung fields (Figure 1a). After a few hours, a follow-up thoracic and abdominal ultrasound (US) was performed, showing a large hypoechoic mass with liver-like parenchyma and vascularization, starting from the fifth intercostal space. The suspicion of a CDHR was raised, and a chest computed tomography (CT) scan was performed, confirming the presence of a posterior CDHR. The hernia defect measured approximately 25 mm, with the cranial ascent of the right liver into the thoracic cavity on the same side, reaching up to the middle-upper third of the right lung field (Figure 1b). There was agenesis of the inferior vena cava in the subhepatic suprarenal segment, with venous return from the lower venous system ensured by slightly ectasic azygos/hemiazygos veins, which drained cranially into the intrathoracic superior vena cava.

Then, an echocardiogram was performed, which showed regular levocardia, normal atrioventricular and ventriculoarterial connections, and regular systemic and pulmonary venous connections.

**Figure 2:**
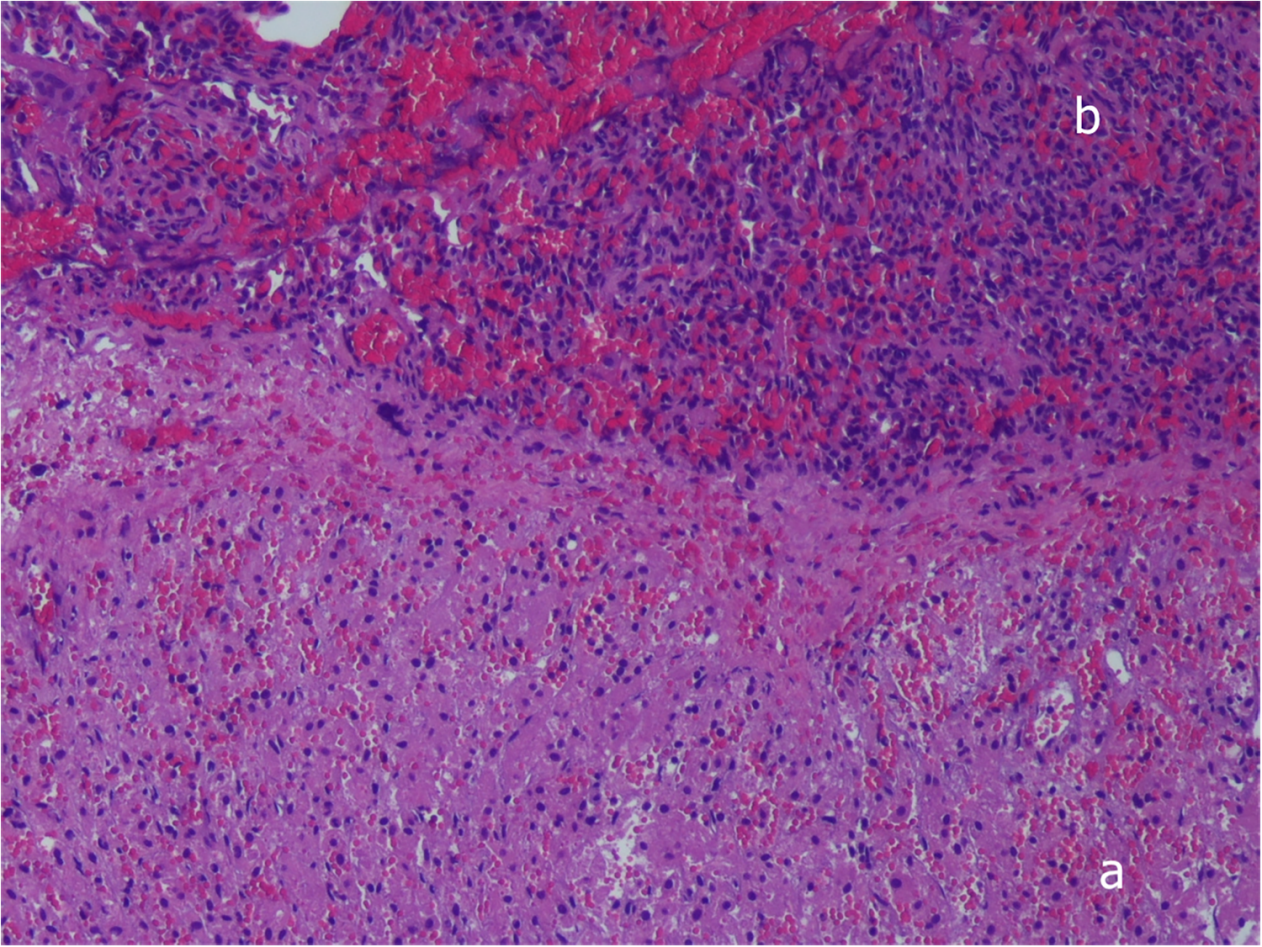
Histopathologic examination, showing complete fusion between liver **(a)** and pulmonary tissue **(b)**, without a plane of separation. Hematoxylin-and-eosin stain, ×10. Written informed consent was obtained from the patient.

Two days after admission to the NICU, at 15 days of life, surgical correction of the diaphragmatic hernia was performed, with the child in stable conditions both from a respiratory point of view (HFOV parameters: MAP 10 cm H2O, FiO2 0.25, RR 10 Hz, Volume guarantee 1.8 ml/Kg requiring *Δ*P of 20–23 cm H2O and corresponding optimal values of pH and blood gases) and hemodynamic (no need for inotropic drugs or pulmonary vasodilators). A right subcostal laparotomy was performed. At exploration no clear plane of cleavage between the diaphragm and the liver could be identified; additionally, the liver appeared firm, and it was impossible to move it towards the abdomen. On the lateral aspect of the liver a flap of tissue was identified with a consistency and color resembling both liver and lung tissue. Bubbles were noted on the thoracic side of this tissue flap. The histological examination of a fragment of this tissue showed hepatic parenchyma connected to lung tissue displaying significant congestion and blood extravasation (Figure 2). The diaphragmatic defect was repaired without separating the lung and the liver: the medial margin of the diaphragm was fixed to the liver surface with three stitches to close the defect. Soon after surgery, the baby developed pulmonary hypertension, leading to endotracheal nitric oxide administration, which was discontinued after 48 h due to resolution of the condition. The remaining postoperative course progressed uneventfully, except for a Staphylococcus epidermidis pneumonia diagnosed 72 h after surgery, which required prolongation of invasive respiratory support for a further 7 days, followed by successful extubation and non-invasive respiratory support for 14 days. Postoperative imaging and laboratory results indicated normal liver function, with no evidence of impairment due to its prior herniation into the thoracic cavity.

At 48 days of life, approximately one month after surgery, an angio-CT scan was performed to obtain a comprehensive assessment of the involved visceral anatomy and associated vascular anomalies. The scan confirmed the presence of a CDHR with intrathoracic herniation of part of the liver, without any vascular communication between the liver and the lung. It also confirmed agenesis of the retrohepatic inferior vena cava, with continuation of the inferior vena cava through the azygos vein.

Considering the achieved clinical stability, no further surgery was performed, and at 58 days of life, the baby was discharged without any respiratory support, with good feeding autonomy, and with the plan to follow a close clinical follow-up.

At 64 days of life, and on the 49th postoperative day, respiratory function tests—Tidal Breathing Flow Volume test and Multiple Breath Nitrogen Washout test with the Exhalyzer D (Ecomedics, Switzerland)—were performed according to published guidelines (7) which showed tachypnea and tidal volume lower than predicted (Table 1).

**Table 1:** Results of the lung function tests.

Lung function tests	9 weeks of age	6 months of age
Tidal volume, ml/kg	6	8
Respiratory rate, breaths per minute	87	52
Inspiratory time, seconds	0.30	0.52
Expiratory time, seconds	0.39	0.62
Time to peak expiratory flow/expiratory time ratio	21.6	18.1
Minute ventilation, ml/kg	502	443
End tidal CO2, %	2.29	3.54
Dead space volume, ml/kg	1.9	3.1
Functional residual capacity, ml/kg	NA	33
Lung clearance index 5	NA	8.53
Lung clearance index 2.5	NA	11.12

Follow-up evaluations are ongoing, and the infant is doing well apart from a mild episode of bronchiolitis which did not require hospitalization. She is getting complementary feeding and palivizumab prophylaxis, and her neurological examination is normal. Growth parameters are satisfactory (last weight was at 2nd percentile, length at 46th percentile, head circumference at 24th percentile). Lung function tests were repeated at 6 months of age: tidal volume was normal indicating catch-up growth, time to peak expiratory flow/expiratory time ratio (tPTEF/tE) was mildly reduced, and lung clearance index (LCI) was high, indicating ventilation inhomogeneity (Table 1).

## Discussion

In our child, the definitive diagnosis of HPF was made during surgery and, because of the abnormal vascular anatomy (Table 2), simple repair of the diaphragmatic defect was chosen, and no attempts were made to separate the liver from the right lung.

**Table 2:** Synopsis of the 38 cases reported in the literature.

Studies	N° of cases	Sex	Age	Mediastinal Shift	Exams	Procedures	Associated anomalies	Outcome	Follow up
Macpherson et al. (31)	3	N/A	N/A	N/A	N/A	N/A	Anomalous systemic circulation to the right lower lobe	N/A	N/A
Katz et al. (24)	1	F	Neonate	N/A		Respect of the fusion and partial separation	CDHR	Died	N/A
Slovis et al. (8)	6	3M 3F	Neonate	4 ipsilateral 1 no mediastinal shift 2 contralateral	X-ray	4 cases: separation of the fusion and diaphragm repair. 2 cases: respect of the fusion and partial repair	5 systemic arterial and venous circulation to a fused lung, 2 left-sided congenital heart disease, 2 PS	2 died, 4 survived	Two 1 year, one 11 years, one no FUP, two N/A
Keller et al. (17)	1	M	Neonate	Toward the lesion	X-ray, lung US, MRI, angiography MRI	Separation of the fusion and diaphragm repair	CDHR	Survived	No FUP
Robertons et al. (20)	1	M	Neonate	N/A	X-ray, lung US	Respect of the fusion and partial separation of the defect	CDHR	Died	N/A
Tanaka et al. (25)	1	F	Neonate	Contralateral	X-ray	Respect of the fusion and partial separation of the defect with hepatic segmentectomy	CDHR	Survived	21 months
Khatwa et al. (23)	1	F	Neonate	Contralateral	X-ray, CT scan	Separation of the fusion and diaphragm repair after partial hepatectomy and a right pneumonectomy	CDHR, absent right PA and right PV, PDA	Died	N/A
Gander et al. (15)	1	M	3 months	Contralateral	X-ray, MRI	Respect of the fusion and partial separation of the defect	CDHR	Survived	7 months
Taide et al. (3)	1	N/A	7 months	No mediastinal shift	X-ray, CT scan	Separation of the fusion and diaphragm repair	CDHR	Survived	18 months
Castle et al. (32)	1	M	Neonate	Contralateral	X-ray, abdominal US	Respect of the fusion and partial repair	Duodenal atresia, imperforate anus, undescended left testicle, bilateral fifth finger clinodactyly	Survived	No FUP
Chandrashekhara et al. (18)	1	M	11 years	Toward the lesion	x-ray, CT scan, MRI, angiography MRI	Separation of the fusion and diaphragm repair after a lower lobectomy	PS	Survived	3 months
Breysem et al. (10)	1	F	Neonate	No mediastinal shift	X-ray, abdominal US, CT angiography	Separation of the fusion and diaphragm repair	Left heart hypoplasia, aortic coarctation, hypoplastic right PA, scimitar syndrome	Died	N/A
Lin et al. (14)	3	1 M 1 F	3 moths, 6 years	N/A	CT scan, HA	1. Hepatic pulmonary effusion was repaired and the sequestration resected. 2. Right lower lobe partial pneumectomy with shaving of the superior surface of the liver and diaphragmatic repair. 3. No surgical intervention, diagnosed by autopsy	2 CDHR, 1 Pentalogy of Cantrell	2 survived, 1 died before the operation	N/A
Hamilton et al. (13)	1	M	3 moths	No mediastinal shift	X-ray, CT scan	Separation of the fusion and diaphragm repair	Anomalous right pulmonary venous return, azygos continuation of the inferior vena cava	Died	N/A
Saurabh et al. (21)	1	N/A	Neonate	No mediastinal shift	X-ray, CT scan	Respect of the fusion and partial separation of the defect	CDHR, thumb and index finger syndactyly, multiple clefts in the vertebrae	Died	N/A
D.Olenik et al. (9)	1	M	Neonate	Contralateral	X-ray, HA	Separation of the fusion and diaphragm repair	CDHR	Survived	No FUP
Laamiri et al. (19)	1	M	Neonate	Contralateral	X-ray	Respect of the fusion and partial separation of the defect	CDHR	Died	N/A
Jain et al. (5)	1	F	2 moths	Ipsilateral	X-ray	Separation of the fusion and diaphragm repair after a lower lobectomy	CDHR and PS	Died	N/A
Takezoe et al. (11)	1	N/A	Neonate	No mediastinal shift	Fetal MRI, x-ray, abdominal US, CT scan	Separation of the fusion and diaphragm repair after a lower lobectomy	CDHR	Survived	N/A
Almaramhy et al. (16)	1	F	Neonate	Contralateral	X-ray, abdominal US	Respect of the fusion and partial separation of the defect	CDHR	Died	N/A
Kerkeni et al. (12)	1	M	Neonate	Contralateral	X-ray, CT scan	Separation of the fusion and diaphragm repair	Dilated right atrium and ventricle, PDA, hypoplastic right PA	Died	N/A
Bawazir et al. (26)	1	M	5 weeks	No mediastinal shift	X-ray, CT scan, angiography	Respect of the fusion and partial separation of the defect	Bilateral diaphragmatic hernia, scimitar syndrome, interrupted vena cava, PDA, ventricular septal defect, anomalous pulmonary venous drainage, PS	Survived	9 months
Patel et al. (27)	1	F	Neonate	Contralateral	X-ray, Ct scan	Separation of the fusion and diaphragm repair	Hypoplastic right PA	Survived	5 months
Oudtshoorn et al. (28)	1	N/A	Neonate	No mediastinal shift	X-ray, CT scan, angiography	Separation of the fusion and diaphragm repair	PS, aortic coarctation, PDA, right ventricular hypertrophy	Survived	6 weeks
Yewei Xie et al. (29)	1	M	4 months	Contralateral	X-ray, CT scan, HA	Separation of the fusion and diaphragm repair	Scimitar syndrome	Survived	No FUP
Clemente et al. (30)	1	M	2 years	No mediastinal shift	X-ray, CCT, CMR	Separation of the fusion and diaphragm repair	Total anomalous right pulmonary venous return, right PH, scimitar syndrome, hypoplastic right PA	Survived	No FUP
Alzaiem et al. (4)	2	2 M	Neonate	No mediastinal shift	X-ray, CT scan	1. Respect of the fusion, partial separation of the defect, partial repair of the defect. 2. separation of the fusion and diaphragm repair	PA hypoplasia, PH	1 died, 1 survived	1 N/A, 1 no FUP
Alomar et al. (1)	1	M	Neonate	Contralateral	X-ray, CT scan	Separation of the fusion and diaphragm repair	Multiple vascular abnormalities	Died	N/A

CCT, cardiac computed tomography; CDHR, right sided congenital diaphragmatic hernia; CMR, cardiac magnetic resonance; CT, computed tomography; FUP, follow-up; HA, histological analysis; MRI, magnetic resonance imaging; N/A, not applicable; PA, pulmonary artery; PDA, patent ductus arteriosus; PH, pulmonary hypoplasia; PS, pulmonary sequestration; PV, pulmonary vein; US, ultrasound.

Our approach was successful, as our patient not only survived the procedure but also showed favorable cardiorespiratory adaptation, consistent growth, and regular neurodevelopment, according to follow-up data, available at six months of life.

The optimal management of this condition is still not clear, given the limited number of documented cases in literature (Table 2). Attempts to separate the liver from the lung parenchyma can be challenging and may require partial pneumonectomy or atypical hepatic resection in some cases. By restoring negative intrathoracic pressure, this procedure should enhance lung development in the affected side and restrict the entry of hepatic tissue into the thoracic cavity (8).

Several surgical approaches have been described for the surgical treatment of HPF. Some surgeons have attempted complete organ separation through resection of the involved tissues (partial hepatectomy or pulmonary lobectomy) (5, 9–14), while others have opted for partial separation and suturing of the diaphragm to the remaining fusion margins (4, 15–21).

A 2019 review by Ferguson described nine cases of HPF identified in the Congenital Diaphragmatic Hernia Registry. Among the reported cases, partial separation of pulmonary and hepatic parenchyma was performed in 6 patients, and among them, one patient did not survive. Complete separation was performed in 2 cases, but both patients did not survive, one due to pulmonary hypertension and the other due to postoperative hemorrhage and renal failure. Finally, separation was not attempted in 1 case, and the surgeon chose to plicate the pleura and peritoneum that were present around the area of fusion. The patient survived to hospital discharge but remained ventilator dependent, and she ultimately expired (6).

In a recent study by Terp et al. (22), complete separation of the lung and liver was possible due to prenatal identification of the anomaly and preoperative characterization of vascular abnormalities. The importance of preoperative diagnosis has been also highlighted by Keller et al. (17), who utilized preoperative chest x-ray, thoracic US, and magnetic resonance imaging (MRI) to establish the diagnosis of CDHR with HPF. In this case, diagnostic and therapeutic cardiac catheterization and preoperative CT were also found to be very useful for preparation and surgical planning.

However, HPF can be missed prenatally and most of the time, it is not diagnosed until surgical exploration (9). HPF, as in our infant, can be asymptomatic at birth or present with cyanosis and respiratory distress. Subsequently, the diagnosis may be incidental, or the most frequent manifestations include recurrent respiratory infections, pleural effusions, and mediastinal compression.

The diagnosis of HPF should be considered when thoracic US or chest x-ray reveals the presence of an opacity in the right hemidiaphragm without mass effect, such as contralateral mediastinal shift or lung compression, due to pulmonary hypoplasia (15). If these findings are associated with cardiac or vascular malformations, they further support the diagnostic suspicion of HPF (5). Exceptions to the above description of HPF can occur, as contralateral or ipsilateral mediastinal shift primarily depends on the size of the diaphragmatic defect and the amount of herniated viscera, regardless of the presence of HPF (16).

The preoperative diagnostic definition is important, above all, because HPF can be associated with cardiac or vascular defects in 10%–30% of patients (4). The association of HPF with cardiac and vascular anomalies can be partially explained by the fact that the hepatic diverticulum, septum transversum, and aortopulmonary septum form near each other during the 4th and 6th gestational week (8).

Among the cases of HPF reported by Ferguson et al., 60% exhibited aberrant right pulmonary vascularization. The most common anomalies include: hypoplastic right pulmonary artery, abnormal pulmonary venous return, inferior vena cava abnormalities (hypoplasia, partial absence of the vena cava, anomalous drainage of the suprahepatic veins), and pulmonary veins that drain into the suprahepatic veins (6).

Due to the uncommon occurrence and anatomical complexity of the condition, some authors suggest that a comprehensive preoperative evaluation with MRI or CT with multiplanar and 3D reconstruction would be beneficial in cases where there is suspicion of HPF. These imaging modalities would enable a comprehensive assessment of the visceral anatomy and provide the most accurate mapping of vascular and bronchial structures (17, 23).

Mortality among cases of HPF is high, and often patients die during the perioperative period. Mortality is mainly related to postoperative complications such as pulmonary hypoplasia, respiratory failure, persistent pulmonary hypertension, right heart failure, congenital heart diseases, and inferior vena cava thrombosis (9, 15).

## Conclusion

Our clinical report and the literature review raise several important observations: (a) prenatal diagnosis is crucial, and suspicion should be raised in all cases of CDHR prenatally diagnosed; (b) preoperative diagnosis is not always feasible but the absence of contralateral mediastinal shift or the presence of rightward mediastinum can act as red flags, prompting additional investigations such as MRI, CT scan, or cardiac catheterization. These examinations allow for a thorough evaluation of the malformation's anatomical aspects and associated vascular abnormalities, to plan the optimal surgical approach.

In our infant, in the absence of prenatal diagnosis, complete separation did not appear to be ideal, given the presence of vascular anomalies that could interfere with perfusion/venous drainage of the liver ant the lung after separation. The adopted surgical management strongly suggests that when the diagnosis is made intraoperatively and detailed knowledge of the vascularization is lacking, partial separation of the viscera, preserving the medial hepatopulmonary fusion and suturing the diaphragm, is the successful approach. One of the strengths of our work is the provision of serial functional respiratory evaluations during the follow-up of the presented patient; these data support the feasibility of our approach and provide further details about lung adaptations in hepatopulmonary fusion. We will continue to follow-up the child in the next years by evaluating respiratory and neurodevelopmental function.

## Data Availability

The raw data supporting the conclusions of this article will be made available by the authors, without undue reservation.
